# Proof of concept: prognostic value of the plasmatic concentration of circulating cell free DNA in desmoid tumors using ddPCR

**DOI:** 10.18632/oncotarget.24817

**Published:** 2018-04-06

**Authors:** Nicolas Macagno, Frédéric Fina, Nicolas Penel, Corinne Bouvier, Isabelle Nanni, Florence Duffaud, Raquel Rouah, Bruno Lacarelle, L'houcine Ouafik, Sylvie Bonvalot, Sébastien Salas

**Affiliations:** ^1^ Department of Pathology, Assistance Publique Hôpitaux de Marseille Timone Hospital, Marseille, France; ^2^ Aix-Marseille University, Medical Faculty, CRO2, UMR 911 (Equipe IV), Marseille, France; ^3^ ID-Solutions, Grabels, France; ^4^ Department of General Oncology, Oscar Lambret Center, Lille, France; ^5^ Department of Molecular Oncology, Assistance Publique Hôpitaux de Marseille, Marseille, France; ^6^ Department of Oncology, Assistance Publique Hôpitaux de Marseille Timone Hospital, Marseille, France; ^7^ Aix-Marseille University, Medical Faculty, Marseille, France; ^8^ Department of Medical Biology, Assistance Publique Hôpitaux de Marseille Timone Hospital, Marseille, France; ^9^ Department of Surgery, Institut Curie, PSL Univeristy, Paris, France

**Keywords:** ddPCR, cfDNA, CTNNB1, desmoid, prognosis

## Abstract

Since desmoid tumors (DT) exhibit an unpredictable clinical course, with stabilization and/or spontaneous regression, an initial “wait-and-see” policy is the new standard of care–thus, the actual challenge is to identify early factors of progression.

We present a method of detection of *CTNNB1* mutations using a targeted digital droplet PCR (ddPCR) on cell-free DNA (cfDNA) extracted from blood samples of 31 DT patients. Furthermore, we analyzed the correlation between DT evolution and plasmatic concentration of total and mutated cfDNA at the time of diagnosis.

Circulating copies of *CTNNB1* mutants (ctDNA) were detected in the plasma of 6 patients (33%) but their concentration was not correlated with evolution of the tumor. Concentration of total cfDNA was higher in the plasma of patients with progressive desmoids (*p* = 0,0009). Using a threshold <900 copies/mL of plasma to detect indolent desmoid and a threshold >1375, it was possible to predict desmoid evolution for 65% of patients by measuring the quantity of circulating DNA in their plasma as early as the time of diagnosis.

Albeit showing that the detection of *CTNNB1* mutants is possible in the plasma of patients harboring a desmoid tumor, the results of this preliminary study raise the hypothesis that most of the circulating DNA detected in their plasma is derived from non-neoplastic cells, most likely normal neighboring tissues being actively invaded. Our results open the perspective of using cfDNA as a biomarker to predict prognosis at the time of diagnosis and assess tumor dynamics to optimize the treatment strategy.

## INTRODUCTION

Desmoid tumors (DT) are rare mesenchymal neoplasms characterized by a high capacity to invade neighboring tissues. Albeit DT do not metastasize, this propensity to invasiveness may lead to iterative recurrences following surgical excision, with variable local failure rates [1] despite clear surgical margins [2]. A wait-and-see management strategy has been proposed following the description of long term progression arrest, spontaneous regression [3, 4] and even tumor growth reactivation triggered by surgery [5–10] : this strategy is now the standard of care. Activating alterations of the wnt/b-catenin pathway are present in 95% of DTs and mostly involves *CTNNB1* (*Cadherin-Associated Protein (Catenin) Beta 1)* or *APC* mutations [11]. In >85% of sporadic DTs, three different types of missense mutation occur recurrently in exon 3 of *CTNNB1*, involving codons 41 or 45. These codons encode respectively the threonine or serine involved in the ubiquitin-mediated degradation of b-catenin, thus preventing it from degradation and increasing its half-life. The most common mutations are represented by p.Thr41Ala (59%), followed by p.Ser45Phe (22%) and p.Ser45Pro (12%) [12–17]. Three large retrospective studies found that p.Ser45Phe *CTNNB1* mutation is a predominant risk factor for local recurrence after curative-intent surgery of primary DT [14, 18, 19]. In a small minority of patients, desmoids result from germline or sporadic loss of *APC*. APC negatively regulates b-catenin stability and loss of APC leads to activation of b-catenin. Currently, one of the caveat of this watchful waiting strategy is still the lack of other validated biological criteria to assess DT aggressiveness.

Circulating cell-free DNA (cfDNA) represent extracellular DNA that circulate in the bloodstream of healthy individuals in low concentration. Albeit their precise origin is still debated, several conditions such as pregnancy, inflammation, autoimmune disorders, traumatism, neoplasia or even intensive physical exercise trigger its release in the bloodstream [20–22]. There is a great enthusiasm in the field of oncology as many applications of liquid biopsies using cfDNA have been reported following the description of targetable activating *EGFR* mutations detected in cfDNA [23]. Data concerning cfDNA in low-grade mesenchymal tumors are nevertheless limited to a single report by Maier *et al.* in which detection of cfDNA harboring GIST-specific mutations was possible [24]. The possibility to detect tumor-specific mutations in cfDNA DTs has not yet been documented.

Digital-droplet PCR (ddPCR) is a very high sensitivity technique that has opened new perspectives in detecting very low concentration of mutated DNA [25, 26] and calculation of absolute cfDNA plasmatic concentration.

We present here a method of detection of DT specific *CTNNB1* mutations using a targeted strategy ddPCR on cfDNA extracted from blood samples and the correlation of total plasmatic cfDNA concentration with evolution of the tumor.

## RESULTS

### Patients

From 2015 to 2016, 31 patients participated in the study, follow-up was available for all patients with a median follow-up of 7 months (range: 0–17 months) (Table 1). Seven patients (22.5%) had a progressive tumor (median time to evolution = 7 months), 17 patients (55%) had stable/non-progressive disease (median follow-up = 6,7 months) and 7 patients (22.5%) harbored a regressive DT (median time to regression = 8 months).

**Table 1:** Clinico-pathological and molecular characteristics of the 31 patients

**Age:**	38 (23–76)
**Sex:**	
Female	21 (67.7)
Male	10 (32.2)
**Location:**	
Abdominal wall	13 (41.9)
Intra-abdominal	2 (6.4)
Extra-abdominal	16 (51.6)
**Number of tumors:**	
Multiple	4 (12.9)
Unique	27 (87.1)
**Size of tumor:**	57.5 (20–160)
**Management:**	
Treatment	8 (25.8)
Wait and See	23 (74.2)
**Biological behavior:**	
Progressive	7 (22.6)
Non-progressive	17 (54.8)
Spontaneous regression	7 (22.6)
**Mutational status (FFPE)**	
T41A	18 (72)
S45F	6 (24)
S45P	1 (4)
APC	2 (6.5)
Wild-type	1 (4)
Undetermined	3 (8)

On the biopsy specimen, 25 DTs (80%) harbored mutations of *CTNNB1* (18 p.Thr41Ala (72%), 6 p.Ser45Phe (24%) and 1 p.Ser45Pro (4%)), 1 DT (3%) was wild-type, and the molecular status of 3 (9%) DTs could not be determined on biopsy material. The two other patients harbored (6%) germinal mutations for *APC* (Supplementary Figure 1). Six healthy volunteers were sampled after informed consent to assess their plasmatic concentration of cfDNA (median age: 29 y/o).

### ddPCR™ quality control results

#### Limit of blank (LOB) and limit of detection (LOD)

#### (Supplementary Figures 2, 3 and Supplementary Table 1)

The minimum fluorescence channel amplitude for FAM to consider a droplet as positive was 2000 for all systems. No false positive droplets were detected for about 2050 ng (615,000 copies) of DNA analyzed on 41 wells containing about 600,000 total droplets. The limit of detection for assays was defined according to Bio-Rad which recommends validating the results with more than 2 FAM positive droplets. Therefore, in case of 3 or more FAM positive droplets, doubly positive droplets (brown, FAM+HEX. Supplementary Figure 2A) were also integrated into the calculations. The 6 dedicated negative control wells had no positive FAM droplets. For the 3 assays, we detected 4 positive wild-type droplets (HEX) (0.09 copies/μl ddPCR) on average for the 55,000 total test droplets. The limit of detection (LOD) for wild-type DNA (Hex) was obtained by multiplying by 3 the LOB. Thus, the samples were included in the analysis with at least an average of 12 wild-type positive droplets per wells. Calculated maximal sensitivity mean for the system was 0,541% (25th: 1,185 – 75th : 0,649).

### Descriptive statistics of ddPCR data

The results for each patient (*n* = 31) were obtained from a median of 76623 total droplets (97.1% CI: 65544 to 88203; 0th: 23820; 25th: 60661, 50th: 76623, 75th: 90366, 100th: 106776). The median of cfDNA copies/μl ddPCR was 10.50 (97,1% CI: 4.16 to 13.00; 0th: 1.13; 25th: 3.85; 50th: 10.50; 75th: 13.48; 100th: 23.50). Concerning the values of normalized cfDNA per ml of plasma, the median was 900 copies/ml (97.1% CI: 346 to 1083.; 0th: 94; 25th: 321; 50th: 875; 75th: 1123; 100th: 1958) corresponding to a median of 2.9 ng/ml plasma for the human diploid genome.

#### Mutated cfDNA cases

Concordant p.Thr41Ala *CTNNB1* mutants were detected in the cfDNA extracted from the plasma of 6/18 patients (33%). The presence of circulating concordant or discordant mutants could not be validated for the other patients. The fractional abundance for p.Thr41Ala ranged from 0.31% to 3,3% in cfDNA (Supplementary Table 1). These 6 patients with a detectable *CTNNB1* mutation (ctDNA) in the plasma encompassed 2 stable, 2 progressive and 1 self-regressive desmoid (Supplementary Table 4). For these patients, there was no statistical correlation between the concentration of circulating mutant copies of *CTNNB1* and evolution of the desmoid, nor between the concentration of total cfDNA and circulating *CTNNB1* mutants.

### Total cfDNA concentration and clinical variables (Figure 1)

**Figure 1:**
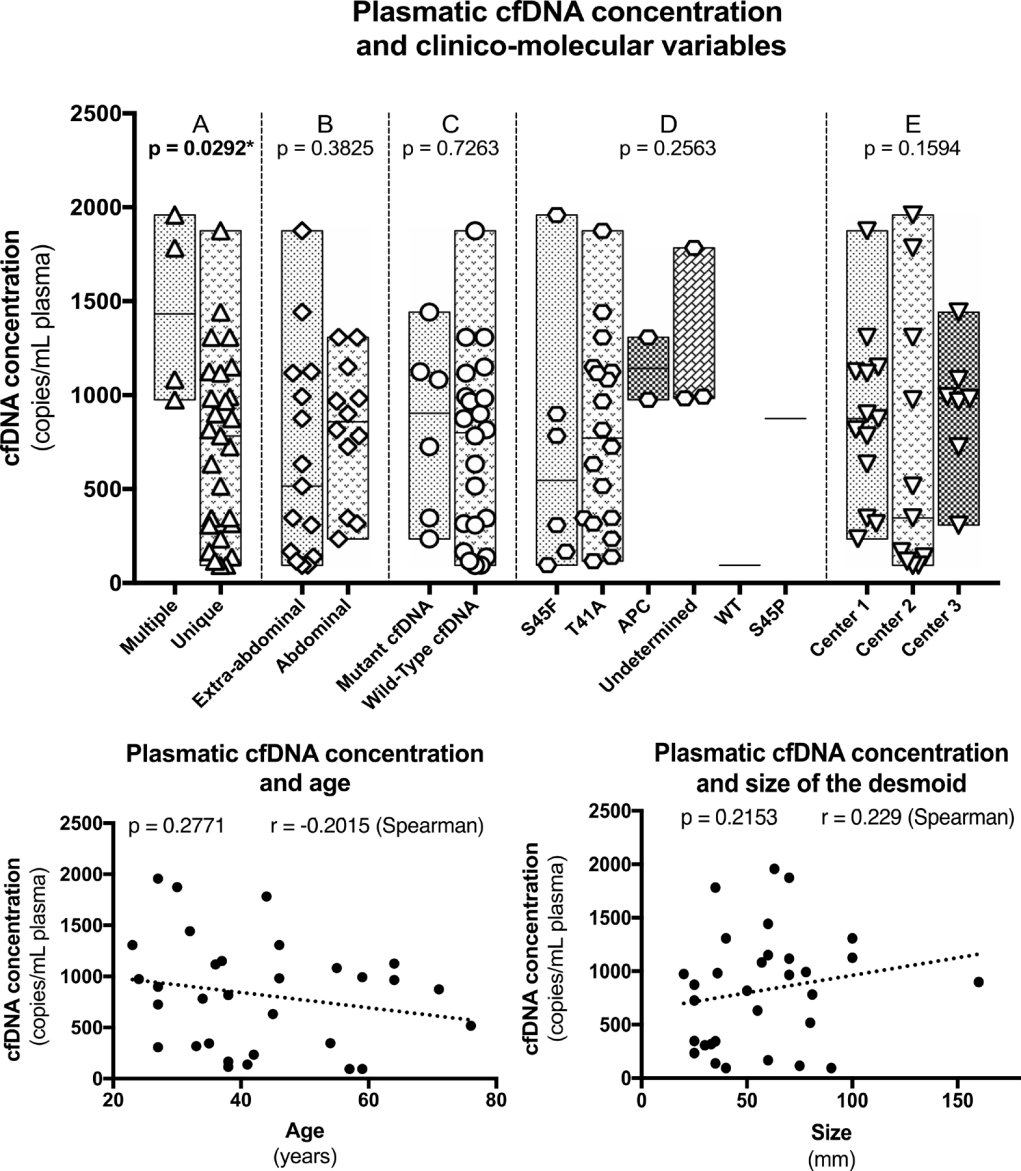
Correlation between cfDNA concentration and the different clinico-pathological variables

Since there was no statistical correlation between the plasmatic concentration of *CTNNB1* mutants and evolution, we focused on total cfDNA concentrations. The plasmatic concentration of total cfDNA was not correlated with age (*p* = 0.2754 – Spearman), size of the tumor (*p* = 0,1226 – Spearman), abdominal or extra-abdominal location (*p* = 0,3825 – Mann–Whitney), *CTNNB1* mutations type (*p* = 0,2563 – Mann–Whitney), presence or absence of a detectable *CTNNB1* mutant in cfDNA (*p* = 0,7263 – Mann–Whitney) and between the different institutions from which the samples were collected (*p* = 0,1594 – Mann–Whitney), Figure 1.

**Figure 2:**
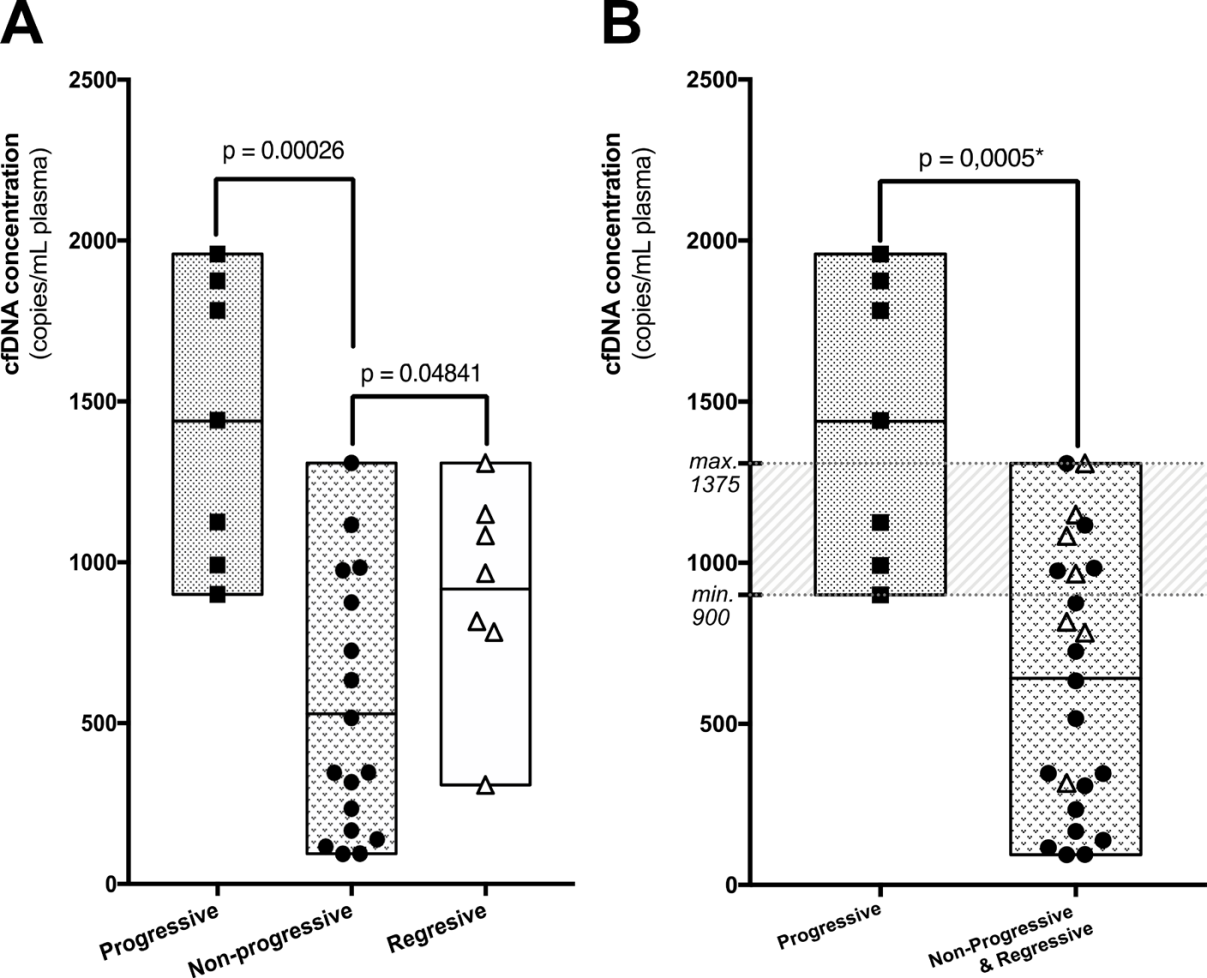
(**A**) Plasmatic cfDNA concentrations measured at the time of diagnosis for patients with a desmoid that progressed (square), self-regressed (triangle) or remained stable (circle) during follow-up. (**B**) Same representation after merging tumors that are managed clinically similarly. A significant number of cases carry overlapping, not informative, concentrations of cfDNA (grey zone). However, using two thresholds, cfDNA levels predicted accurately the evolution of desmoids for 65% of patients: a cfDNA concentration >1375 copies/mL of plasma was always indicative of a progressive desmoid and a concentration <900 copies/mL was always indicative of a stable or regressive desmoid.

Nonetheless, total cfDNA concentration at the time of diagnosis was higher in patients harboring multiple DTs (*p* = 0,0292 – Mann–Whitney) and more interestingly in patients with a progressive DT during follow-up (*p* = 0,0009 – Mann–Whitney). The concentrations were also different between patients with a progressive, a non-progressive and a self-regressive DT (*p* = 0,0012 – Mann–Whitney) (Figure 2A). Albeit marginal, the concentrations were also different between non-progressive and self-regressive DTs (*p* = 0.04841 – Mann–Whitney). When patients of the self-regressive and stable desmoids were grouped, as they are management in a same manner, the concentrations were different compared to patients with progressive tumors (*p* = 0.0005 – Mann–Whitney), Figure 2 and Table 2.

**Figure 3:**
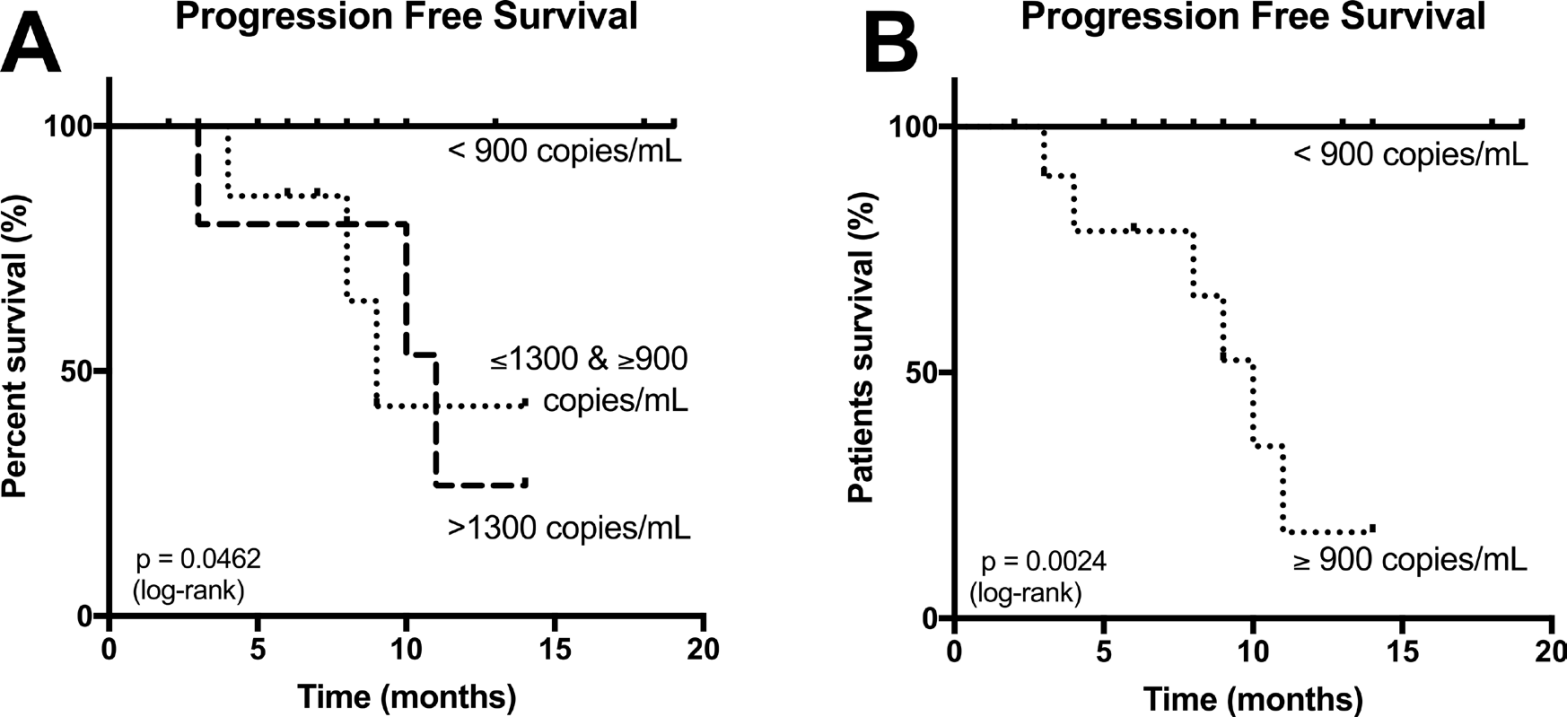
(**A**) Progression free survival (PFS) based on the plasmatic concentration of cfDNA at the time of diagnostic with patients carrying <900 copies/mL of plasma, patients with concentrations within they grey zone, and patients with concentrations >1375 copies/mL of plasma: concentrations superior to 900 copies/mL of plasma were correlated with progression of the desmoid during follow-up. (**B**) Same curve of survival, simplified to display only < or ≥ 900 copies/mL of plasma cut-off.

**Table 2:** Concentration of plasmatic cfDNA according to clinics-molecular variables

	*N*	cfDNA plasmatic concentration (copies/mL)	95% CI	*p*–value (Mann–Whitney test)
**Age:**				0.5011
<38 y/o	16	894	591.1–1197	
>38 y/o	15	745.1	466.8–1023	
**Sex:**				0.9581
Female	21	822.4	387.9–1257	
Male	10	821.7	588.8–1055	
**Location:**				0.3931
Abdominal	14	899.8	655.1–441.3	
Extra-abdominal	17	757.8	1144–1074	
**Number of tumor(s):**				0.0268^*^
Multiple	4	1450	919.1–2235	
Unique	27	728.9	538.6–665.3	
**Size of tumor:**				0.1008
<55 mm	15	659.1	679.1–1270	
>55 mm	16	974.6	395.5–922.6	
**Biological behavior:**				0.0012^*^
Progressive	7	1439	900–1958	
Non-progressive	17	528.7	166.7–875	
Regressive	7	916.7	308.3–1308	
**Mutational status (FFPE)**				0.1689
T41A	18	726.7	134.5–1319	
S45	7	818.2	560–1076	
APC	2	1142	−976–3259	
Wild-type	1	220.4	−1384–1825	
Undetermined	3	1253	111.3–2394	
**Mutational status (cfDNA)**				0.9839
Absence of mutation	25	820.9	591.3–1051	
Mutated cfDNA	6	826	328.2–1324	

Patients with a progressive DT (*N* = 7) displayed at the time of diagnosis a mean of 1439 *CTNNB1^wt^* copies/mL of plasma (CI 95% :900–1958), those with a stable/non-progressive DT (*N* = 17) a mean of 528.7 *CTNNB1^wt^* copies/mL of plasma (CI:95%: 166.7–875) and those with a regressive DT (*N* = 7) a mean of 916.7 *CTNNB1^wt^* copies/mL of plasma (CI 95%: 308–1308). The group of patient harboring regressive and stable/non-progressive (*N* = 24) DTs displayed a mean of 641.8 *CTNNB1^wt^* copies/mL of plasma (CI 95% : 467–816).

**Figure 4:**
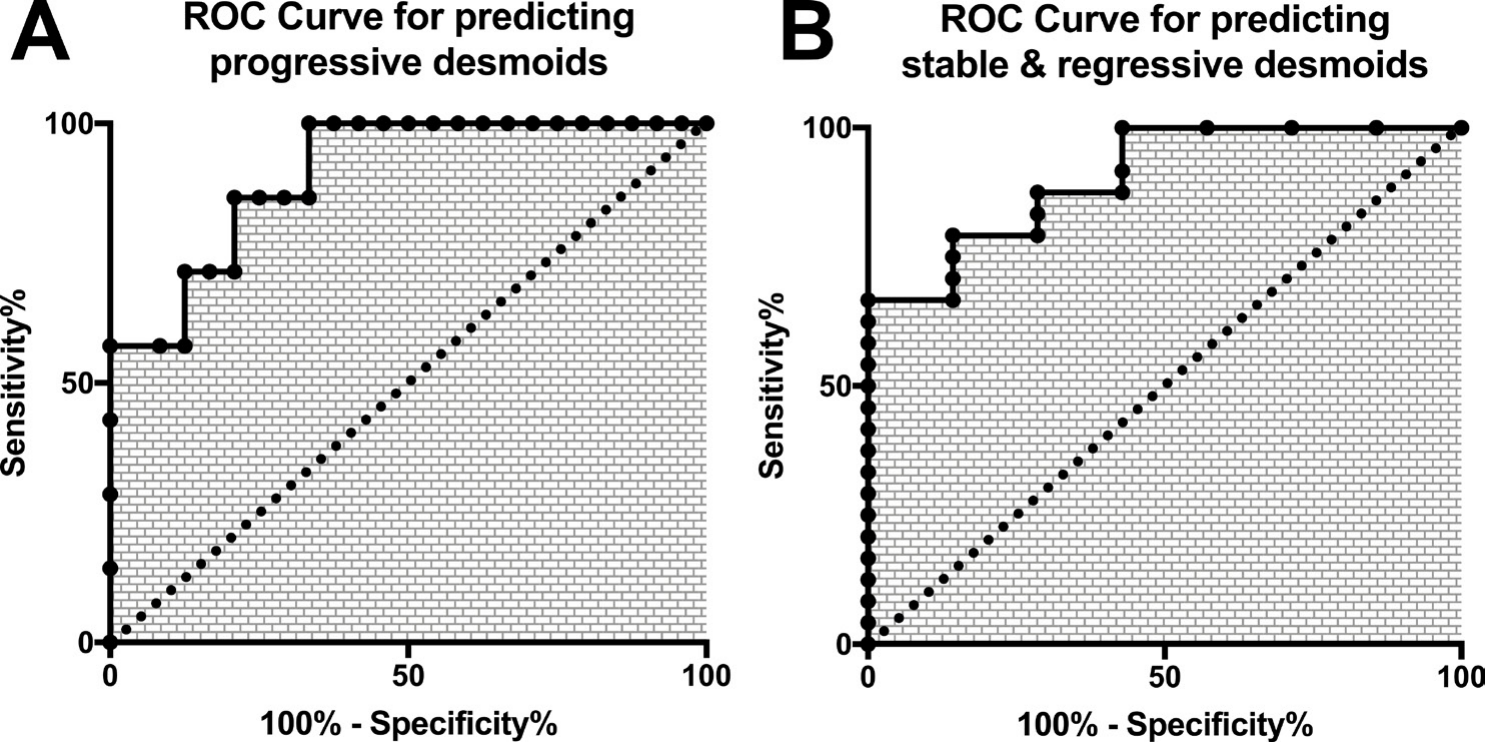
Receptor Operative Characteristics curves of the plasmatic concentration of cfDNA for (**A**) the prediction of progressive (**B**) the prediction of stable/self-regressive desmoids.

In our cohort, the sensitivity to detect progressive desmoids using a threshold ≥900 *CTNNB1^wt^* copies/mL of plasma was 100% (CI 95%: 59–100) and the specificity was 76,5% (CI 95%: 50.1–93.2). However, using solely one single threshold, 8 cases (25%) would have been misclassified as cfDNA concentrations were overlapping between groups, Figure 2B. In order to be clinically relevant and minimize false-positive cases, we calculated the boundaries of this zone for which concentrations carried uncertainty. A threshold <900 *CTNNB1^wt^* copies/mL of plasma had 66% sensitivity and 100% specificity for predicting clinically indolent DTs (stable/non-progressive and regressive DTs) and a threshold >1375 *CTNNB1^wt^* copies/mL of plasma had 57.14% sensitivity and 100% specificity to detect progressive DTs. The receiver operative characteristics curves (ROC) at the time of diagnosis to differentiate “progressive” and “stable/non-progressive + regressive desmoids” patients using cfDNA concentration are displayed in Figure 4 (both: AUC = 0.90; *p* = 0.0013). With this double threshold approach, measuring cfDNA concentration at the time of diagnosis was clinically informative for 65% of cases. The remaining 11 cases (35%) encompassed 3 progressive, 4 regressive and 4 stable DTs.

A concentration ≥900 *CTNNB1^wt^* copies/mL of plasma was detected in all patients with a desmoid that progressed during follow-up. Progression free survival (PFS) curves are displayed in Figure 3, with a median PFS of 10 months. The PFS curve of patients carrying concentrations within the grey zone (≥900 and ≤1375) is also displayed.

Finally, we compared our results with the cfDNA concentration measured in the plasma of 6 healthy volunteers: the median concentration of cfDNA was 415.6 *CTNNB1^wt^* copies/mL of plasma (CI 95%: 294.3– 554.3). Healthy persons carried inferior concentration of cfDNA compared to patients harboring a progressive (*p* = 0.0012 – Mann–Whitney) or a self-regressive desmoid (*p* = 0.0221 – Mann–Whitney). No such difference was stated with patients harboring a stable desmoid (*p* = 0.9729– Mann–Whitney). The complete data of cfDNA for healthy patients is displayed in Supplementary Table 5.

## DISCUSSION

For the first time, to our knowledge, we present a method to detect plasmatic *CTNNB1* concentration and mutation using a targeted ddPCR on cfDNA extracted from blood samples of patients with a DT. More importantly, we have found a correlation between desmoid evolution during follow-up and the concentration of total plasmatic cfDNA at the time of diagnosis. Our findings open the perspective of using cfDNA as an early biomarker to assess DT behavior. This method also opens monitoring perspectives during the wait-and-see strategy and for treatment decision.

Our most interesting finding was that the plasmatic concentration of total cfDNA detected at the time of diagnosis was correlated with disease evolution, independently of the mutational status of *CTNNB1*. This counterintuitive result question the origin of the cfDNA detected. Quantification of cfDNA is a promising technique to monitor tumor dynamics in several kinds of malignancy [27]. The concentration of cfDNA, and particularly the fraction of cfDNA specifically derived from the tumor itself, namely ctDNA, was correlated with tumor burden and stage in several studies [28–32]. Unlike the concentration of total cfDNA, the concentration of specific circulating mutated copies of *CTNNB1* was not correlated with DT evolution albeit such mutant copies were supposedly originating from the neoplastic cells and this counterintuitive result must be balanced with the low number of patients with a detectable circulating mutation of *CTNNB1* in the plasma. Monitoring cfDNA without assessing its neoplastic origin is still a matter of debate. Methods using a panel of different gene mutations [27], comparison of methylation profile and/or fragmentation patterns are promising approaches to improve the specificity of cfDNA and address this issue [33, 34].

However, increasing data encourage the idea that DNA circulation is a non-specific phenomenon. Higher cfDNA concentrations can be detected in different pathological or physiological processes: inflammation, cancer, graft-rejection, infections, aging, pregnancy, intense exercise or stress [35–38]. In cancer specifically, higher plasmatic concentration of total cfDNA carries a prognostic value for colorectal cancer [39, 40] and lymphoma [41]. It may also be predictive of relapse in lung cancer following surgery [42].

In our study, evolution was linked to total cfDNA concentrations, but not with circulating mutant copies of *CTNNB1* concentrations (ctDNA). Albeit the low proportion of patients with detectable ctDNA may result in biases, these results are surprising and troubling. In particular, they raise the hypothesis that the majority of cfDNA detected in patients harboring a desmoid may originate from non-neoplastic cells, either from normal tissue affected by the desmoid growth/invasion or cells composing the desmoid micro-environment. Desmoids are indeed characterized by their high propensity of local invasion and destruction of tissue which is replaced by fibrosis which supports this hypothesis: during such invasion, the invaded normal tissues will undergo inflammatory, atrophic, ischemic, apoptotic or necrotic changes, thus releasing high amount of wild-type DNA into the blood stream. In a molecular study encompassing 117 desmoids being prepared from cryomolds and dissected to remove contaminant, whole-exome data showed that only a minority of reads displayed *CTNNB1* mutation, suggesting that desmoids might be infiltrated by normal of inflammatory cells with the possibility that these accompanying cells may also release wild-type cfDNA. The hypothesis on the origin of cfDNA in desmoids also explains the lack of concordance between cfDNA concentration and tumor size, as cfDNA might in fact reflect better the active invasion of desmoids rather than their size. Albeit size of the tumor was not correlated with concentration of cfDNA or ctDNA, higher concentrations of cfDNA were detected in patients with multiple tumors, a clinical setting which is generally associated with a more aggressive course. Finally, another study by PET/FDG also reported that cfDNA was better correlated to the tumor biological behavior/aggressiveness than the tumor burden [43].

Intermediate cfDNA concentrations were detected for patients harboring self-regressive desmoids: we do not have a proper explanation for this, but the regression phenomenon itself is probably driven by events such as inflammation which is known to be linked to cfDNA release. Finally, a statistical difference was found between the cfDNA concentrations of regressive and stable desmoids, but this difference was marginal and the cohort size was limited, so these results require further validation.

On a genomic point of view, gains or amplifications in neoplastic cells may result in higher concentrations of plasmatic cfDNA. Nevertheless, desmoids are characterized by point-mutation of *CTNNB1* or deletion of *APC* and do not harbor copy number change, gains or amplification as CGH-array data suggest [44–48].

Monitoring a plasmatic biomarker requires defining thresholds. Albeit defining one single threshold of ≥900 DNA copies/mL could predict the progression of DT at the time of diagnosis with a sensitivity of 100% and a specificity of 76.5%, 25% of patients (8 stable/regressive patients) would have been misclassified as they carried concentrations ≥900 DNA copies/mL, uncorrelated to the size of the tumor.

Concomitant disease may explain this phenomenon for 37% of patients: 1 patient also developed a colorectal carcinoma during follow-up, 1 developed a high-grade epithelial dysplasia of the small intestine and 1 presented pain probably linked to inflammation. All of which may have resulted in higher cfDNA. We do not have a clear explanation for the 5 other patients. For them, serial sampling may be required to monitor desmoid activity and accurately predict behavior.

In our cohort, there was an overlap between critical groups. Since basing the clinical decision on values above one single threshold would have misclassified one quarter of cases, we defined a minimum and a maximum threshold for which cfDNA concentrations values would always be clinically informative, Supplementary Tables 2 and 3. All patients with a concentration of plasmatic cfDNA <900 copies/mL (lower threshold) harbored desmoids that remained stable or self-regressed during follow-up and for which keeping-up with the wait-and-see strategy would be relevant. Contrariwise, a plasmatic concentration >1375 copies/mL (upper threshold) was always indicative of a desmoid that would progress during follow-up. At the time of diagnosis, with a simple blood sample, clinicians can use the information provided by the plasmatic concentration of cfDNA to select patients that may beneficiate of a treatment. In this setting, the levels of cfDNA were able to predict the clinical behavior of the desmoid as soon as the first consultation for 65% of patients. The possible integration of testing cfDNA is summarized in Supplementary Figure 4.

To address the problem of uncertainty for the remaining patients carrying concentrations within the “grey” zone, further investigations are needed to validate our results, refine the value of the thresholds and in particular precise the role of serial sampling.

We have also analyzed the plasmatic concentration of cfDNA for 6 healthy volunteers and all carried concentrations inferior to the patients harboring progressive or self-regressive desmoids. Of note, in this preliminary study, the number of patients in this control group was small and matching on age, sex or any other variables affecting cfDNA concentrations could not be performed.

In this feasibility study, we have shown that p.Thr41Ala mutations of *CTNNB1*, which is the most common type of *CTNNB1* mutation driving sporadic DTs, are also detectable in the plasma of patients. Albeit using digital droplet PCR, which is a highly sensitive technique, circulating mutant copies of *CTNNB1* could be detected in only one third of patients. The two other main types of *CTNNB1* mutation could not be detected in the plasma by our ddPCR approach, despite rigorous quality controls. Of note, p.Ser45Phe mutants were detected, but the number of positive droplets was under the required threshold to be considered contributive. Since ddPCR can detect very low fractional abundance mutant alleles, these low detection rates may be relative to the low-grade nature of DTs, which are neoplasms characterized by absence of hypercellularity, necrosis or brisk mitotic activity, events that are thought to trigger the release of DNA in the bloodstream. Indeed, the fractional abundance of *CTNNB1* mutants in cfDNA was low. It has been reported in a large study that DTs could harbor *CTNNB1* mutations at a low allele frequency, with only a minority of reads mutated for *CTNNB1* [11], raising the hypothesis of *CTNNB1* mosaicism in desmoids. Unfortunately, the corresponding rate of mutation in the paraffin blocs could not be centrally analyzed to correlate this phenomenon, but, of note, *CTNNB1* mutations were detected on FFPE by a direct sanger sequencing technique which has an “optimal” sensitivity ranging from 5–10%. Since most of the cases were detected positive on FFPE, we may extrapolate that the lowest rate of mutation in FFPE was at least 5–10% for most patients. Collection of a greater blood volume and optimization of the extraction process may dramatically improve the yield of extraction, increasing the number of detectable cases.

The current limits for the routine use of cfDNA are related to the impossibility of ensuring the neoplastic origin of the DNA analyzed and *CTNNB1* mutations are not specific to desmoids. Other neoplasms and in particular carcinomas, may also harbor mutations of this gene [12]. Of note, the clinical presentation of DT and carcinoma are different. In this context, before using cfDNA as a diagnostic tool, integration with the clinical context, histological examination and molecular biology on the FFPE sample should remain the gold standard. In our cohort, no patient presented another malignancy at the time of inclusion.

The pre-analytical phase is a key step of the analysis. Considering that the pre-analytical phase may affect the yield of extraction and in order to remove the obstacles of using cfDNA as a biomarker, quantification, optimization and standardization are mandatory [49]. This step requires to be perfectly mastered, particularly when tubes with anticoagulant (EDTA) but without a specific stabilizer are used to store the blood samples. The pre-centrifugation conveying delay, the volume of extracted plasma and the choice of the extraction technique influence the quality of the results. One of the first improvement of the pre-analatyical could be using in priority specific collection tubes for cfDNA. Optimizing the volume of eluted DNA analyzed could be another vector of improvement: indeed, the number of droplets dedicated to analysis had a major influence on the variability of quantification. This was especially the case for low DNA concentrations. In a clinical context where assessing a mutation for diagnosis or theranostic purposes is the priority, using half of the volume of eluted DNA (50 μL) may appear prohibitive as it may reduce sensitivity, but, as our results suggest, searching for a specific molecular anomaly might not be the key parameter for desmoids to predict their behavior. The analysis of the impact of the number of wells dedicated to the analysis (Supplementary Figure 3) shows that, for low values (3–4 copies/microliter of ddPCR), 40,000 total events are enough to obtain a standard deviation close to 20%, equivalent to 3 wells in the case of the QX200 (Bio-Rad). In other words, it means that for absolute cfDNA quantification, less wells (and therefore less DNA analyzed) are needed than the number of wells that have been used in this study. Another important step is the extraction, and in particular, monitoring over time cfDNA concentrations could be carried out by considering the extraction step as part of the analytical. Therefore, an external calibrator of DNA compound with an alien sequence could be added to each sample, to monitor the variation of genomic DNA as a function of this external sequence that would undergo all the variations inherent of the different steps of the analytical chain. Using this external calibrator would not only allow the assessment of kinetics between different independent samples but also improve the test to ultimately use it as a biomarker for monitoring purposes. Additionally, the current techniques yield a great volume of elution and therefore the DNA concentration is in low concentration. In the end, only part of the 4 ml of plasmas is analyzed, resulting in reduced sensitivity and specificity. To solve this problem, following the examples of cobas^®^ z480 (Roche) and Idylla™ (Biocartis) in lung or colon cancer, we decided to analyze the clear majority (50%) of the elute DNA extracted from 3–4 ml of plasma, we analyzed 48 μl of DNA with 6 wells per patient. The other advantage of this approach concerned the reduction, as much as possible, of the error rate produced on the concentration expressed in copies/μl of ddPCR™ (Poisson's law, 95% confidence) by analyzing at least 54000 droplets in total, according to the recommendations of Bio-Rad (for which an analysis is valid when 9000 or more events/well are reached).

Our results and the many questions raised are the starting point of a prospective study named ALTITUDE (Clinical trials n° NCT02867033) on desmoid tumors. This study will help confirm the value of monitoring cfDNA at the time of diagnosis and during the wait and see strategy.

## CONCLUSIONS

To conclude, the plasmatic concentrations of cfDNA at the time of diagnosis were correlated with desmoid evolution during the wait-and-see strategy. Mutated copies of *CTNNB1* can be detected in a very low concentration in the plasma of patients harboring desmoid tumors but without being correlated with outcome, thus questioning the exact origin of total cfDNA variation. This study opens the perspective of using cfDNA monitoring to predict desmoid dynamics at the time of diagnosis, during the wait-and-see strategy and following therapy. To confirm these results, we are planning a prospective validation study that will follow clinical and biological best practices.

## MATERIAL AND METHODS

### Patients

Patients were enrolled in the study after informed consent from three different French expert-centers (Lille, Marseille and Paris) during the first consultation following the histological diagnosis of DT, confirmed by assessment of the *CTNNB1* mutational status on the biopsy material when possible either using direct sanger sequencing or High Resolution Melting PCR (HRM-PCR) followed by sanger sequencing. For germline *APC* mutation, sequencing was performed on DNA extracted from peripheral blood cells using bidirectional sanger sequencing of the entire coding region and intron/exon borders [50] or by multiplex ligation-dependent probe amplification (NGS) to detect large deletions/duplications. Progression was defined by the clinical urge to perform a treatment following an increase in tumor volume using RECIST criteria and/or due to pain or loco-regional compression within the first 6 months of follow-up. No patients had a priori history of treatment. Regression was defined by a partial or complete reduction of DT size, assessed clinically or radiologically.

### Methods

Briefly, blood samples were collected from all patients at the time of diagnosis, before any treatment was performed. Blood was centrifuged to separate the plasma from which cfDNA was extracted and entirely used as a template for a ddPCR assay targeted to wild-type and mutant *CTNNB1* (p.Thr41Ala, p.Ser45Pro p.Ser45Phe) in order to calculate the absolute concentration of mutant and wild-type cfDNA copies of *CTNNB1* in the plasma of the patient at the time of diagnosis. Complete ddPCR data is available in Supplementary Table 1. A group of healthy patients was sampled following the same techniques, using EDTA tubes, to assess the concentration of cfDNA in their blood.

### Blood collection and centrifugation procedure

All analyzes were centralized in Marseille. Blood samples were collected locally EDTA tubes (K2EDTA), while the centers of Lille and Paris used cell-free DNA BCT tubes (Streck, USA), a minimum of 10 mL of blood was taken. The centrifugation conditions were identical independently of the type of tube, except for the latency time between sampling and centrifugation which required a delay of less than 4 hours for EDTA tubes: first centrifugation was performed at 1600 g for 10 minutes (15–25° C) followed by aspiration of the plasma avoiding the intermediate phase, plasma was then transferred into a conical tube for a second centrifugation at 4500 g to eliminate any nuclei and whole cells at the bottom of the tube. The plasmas were stored at −80° C before extraction.

### Plasmas volume and DNA extraction procedure

Extraction was carried out using the IDXtract cfDNA kit following the recommendations of the supplier (IDSolutions, France). The extracted plasma volume was in the range of 3 to 4 ml for elution volumes of 100 μl DNA. These volumes were collected in order to normalize results by mL of extracted plasma.

### Digital droplet PCR (ddPCR™)

In ddPCR™, target DNA molecules are distributed across multiple replicate reactions at a level where some reactions may have no DNA template present and others may have one or more template copies present. After amplification to the terminal plateau phase of PCR, reactions containing one or more templates yield positive end-points, whereas those without template remain negative. The number of positive and negative droplets in each reaction is used to calculate the concentration of the target mutated and reference DNA sequences and their respective Poisson-based 95% confidence interval.

ddPCR™ experiments were performed following the recommendations of the supplier (Bio-Rad) for 8 μL of cfDNA of template. PrimePCR™ ddPCR™ mutation assay (Bio-Rad) was used for *CTNNB1* p.Thr41Ala (dHsaCP2000548, FAM_IowaBlack) and p.Ser45Phe (dHsaCP2000117, FAM_IowaBlack) mutations and *CTNNB1* WT assay, dHsaCP2000548, HEX_IowaBlack and dHsaCP2000117, HEX_IowaBlack for p.Thr41Ala et p.Ser45Phe respectively. Home specific design for p.Ser45Pro was used (S45Pfor: CCATTCTGGTGCCACT; S45Prev: ATCCTCTTCCTCAGGATTG; S45^wt^: ACTCAGAGaAGGAGCTGT HEX_IowaBlack and S45P: ACTCAGAGgAGGAGCTG FAM_IowaBlack) The PCR reaction conditions were the same as for the PrimePCR™ ddPCR™ mutation assay. The length of amplicon was 65 bp for p.Thr41Ala, 62 bp for p.Ser45Phe and 66 bp for p.Ser45Pro.

After homogenization, the PCR reaction mixture and droplet-generation oil for probes were loaded into an eight-channel droplet generator cartridge (Bio-Rad Laboratories). The PCR reaction mixtures were partitioned into an emulsion of approximately 15,000 droplets (~1 nL per droplet) which were manually transferred to a 96-wells PCR plate. The PCR plate was heat-sealed and placed in a conventional thermal cycler. Following the PCR, the 96-wells plate was loaded on a QX100 droplet reader (Bio-Rad Laboratories), analysis of the ddPCR™ data was performed with QuantaSoft software (version 1.7.4.0917) which analyzes each droplet individually using a two-color detection system (set to detect FAM or HEX dyes). The absolute quantification of DNA (Poisson Law, 95% confidence) is directly dependent on the number of accepted droplets (positive plus negative) and the DNA quantity analyzed.

### Limit of blank (LoB) and limit of detection (LoD)

When measuring rare events, it is necessary to define the background noise generated by the system in the presence of wild-type DNA and to define the height expected for the signal.

To determine the susceptibility of auto-hydrolysis or aspecific hybridization for every primer-probe couple (p.Thr41Ala, p.Ser45Pro, p.Ser45Phe and their respective *CTNNB1* wild-type probe) which may result in fake-positive droplet signal, each primer-probe couple was tested on DNA extracted from human placenta harboring wild-type *CTNNB1* gene (6 ng/uL per well), with positive controls composed of DNA extracted from FFPE in which a desmoid tumor with a known mutation of *CTNNB1* was embedded. The maximum fluorescence detected for each mutation-specific primer-probe couple permitted to set a minimum threshold of fluorescence amplitude to consider a droplet positive. It also permitted to determine the number of false positive chambers that must be considered to define the limit of detection. Placenta DNA was used as a wild-type control, 41 wells containing 50 ng of genomic DNA, one well is dedicated to positive control and 7 wells for negative control (no DNA), were analyzed for each ddPCR™ system.

### cfDNA specific ddPCR™ analytical modalities

Droplets are produced in series of eight: 1 positive control, 1 negative control and the remaining 6 wells are dedicated to the same patient. The concentrations of cfDNA wild-type were expressed as cfDNA copies, normalized by ml of extracted plasma (copies/ml of plasma).

### Statistics

The chi-square test (or Fisher's exact test when one subgroup was *n* < 5) for categorical variables and Mann–Whitney *U*-test for continuous variables were used. All statistical tests were two sided, and the threshold for statistical significance was *p* = 0,05. The results are reported as two-sided *P*-values with 95% confidence intervals (95% CI). Variables assuming a non-Gaussian distribution were computed according to the nonparametric Spearman correlation. The droplet reader generated 95% CI interval according to Poisson's law. Analyses were conducted with Analyze-It (Analyze-it Software), Prism (Graphpad Software) and Quantasoft (Bio-Rad).

NM and FF wrote the manuscript, performed the experiments and the statistical analysis. RR performed the experiments. FD, NP, SB and SS provided patients samples and follow-up data. IN managed the samples and patient's data. NP, SB, LO and BL reviewed the manuscript. SS supervised the study.

## SUPPLEMENTARY MATERIALS FIGURES AND TABLES








