# Supplementary material for: Proof of concept: prognostic value of the plasmatic concentration of circulating cell free DNA in desmoid tumors using ddPCR
Source: Oncotarget. 2018 Apr 6;9(26):18296–308. doi: 10.18632/oncotarget.24817 (PMC5915073; doi:10.18632/oncotarget.24817)
Supplement: Supplementary file 1 [file oncotarget-09-18296-s001.pdf]

Proof of concept: prognostic value of the plasmatic concentration of circulating cell free DNA in desmoid tumors using ddPCR

SUPPLEMENTARY MATERIALS

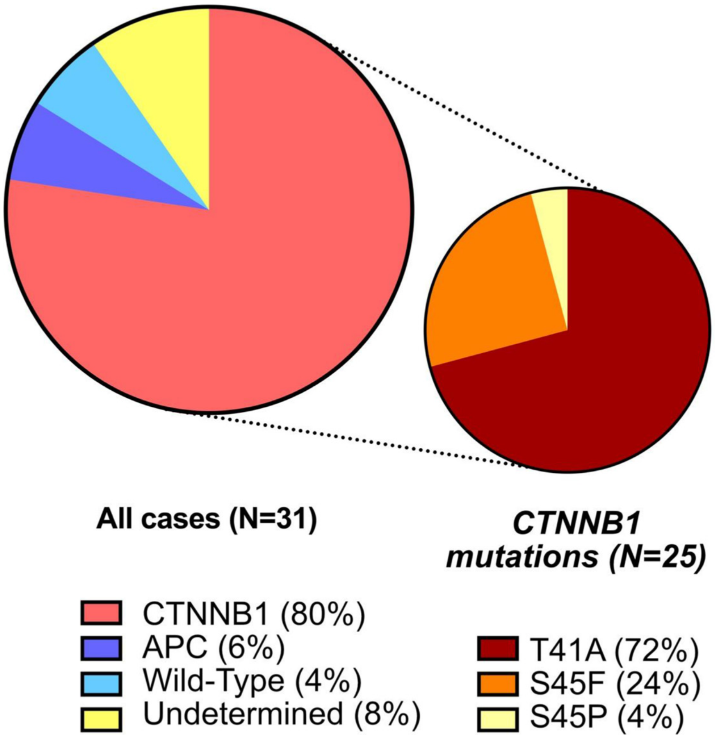

Supplementary Figure 1: Mutational status of the 31 patients' desmoids.

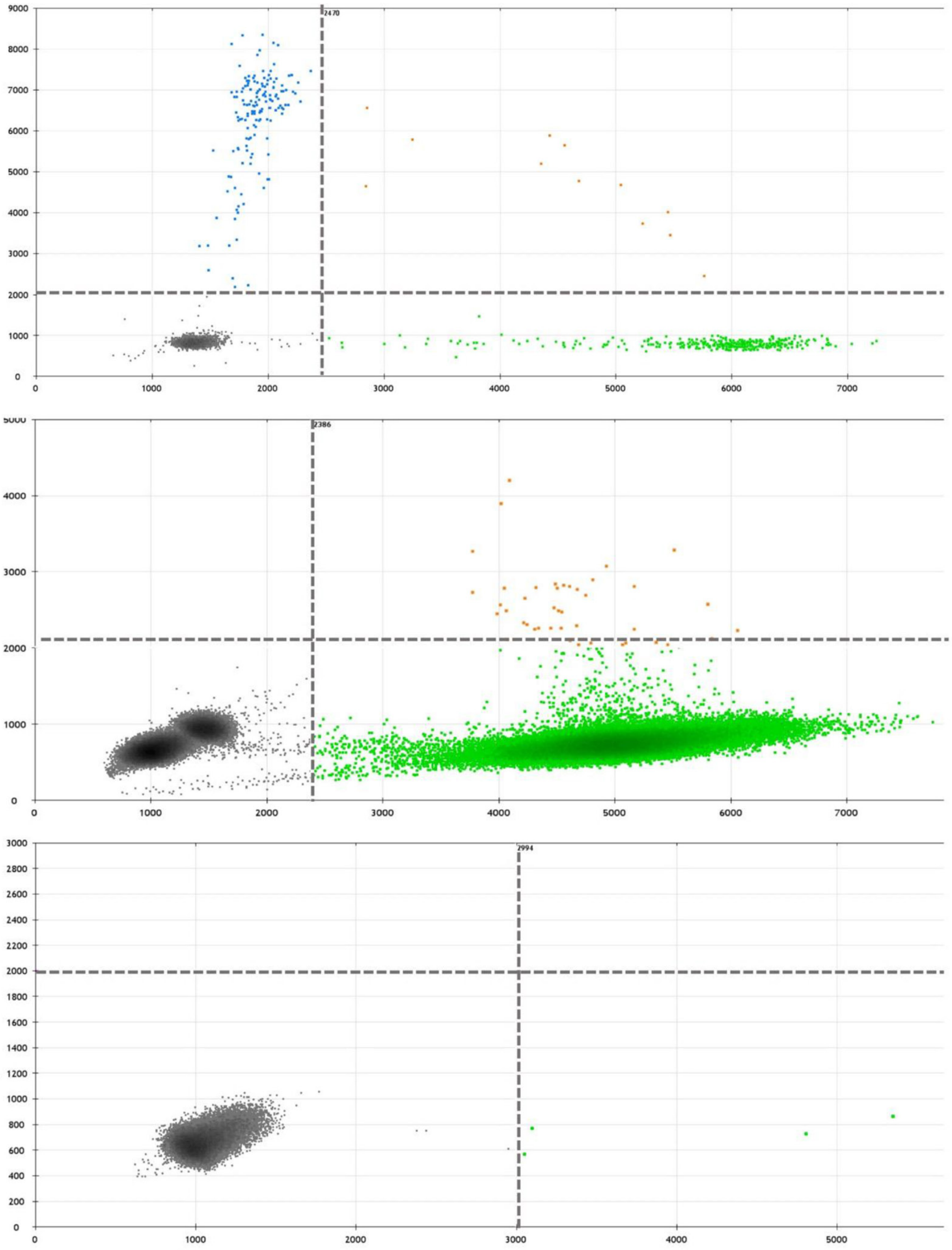

Supplementary Figure 2: Examples of ddPCR analysis, the corresponding area of interest to consider positive droplets with the HEX (wt-*CTNNB1*) or FAM (mutated-*CTNNB1*) signals are separated by the dashed lines, which correspond to the thresholds (2000 for FAM, between 2000-3000 for HEX). (**A**) Top figure: positive control displaying high-concentration of mutated-CTNNB1: a cloud of FAM composed of many positive droplets (corresponding to positive fluorescence signal for mutated copies of CTNNB1) is present in the upper-left corner. The HEX cloud (corresponding to wild-type copies of *CTNNB1*) is present in the lower-right corner. All spots in between in the upper-right corner are doubly marked and the remaining spots in the lower-left corner are negative droplets. The sum of HEX, FAM, and doubly spots represents the absolute amount of DNA analyzed. (**B**) Center figure: case with high concentration of cfDNA without mutant for *CTNNB1*: there are no FAM positive droplets, a great concentration of HEX-positive droplets, and some doubly droplets are present (doubly spots cannot be considered positive without at least 2 accompanying FAM-positive droplets). The total amount of DNA is greater than the previous example as the number of HEX-positive droplets is higher). (**C**) Bottom figure: case with very low concentration of cfDNA without mutant for CTNNB1: only rare HEX positive droplets are present and corresponds to very low non-mutated DNA concentration.

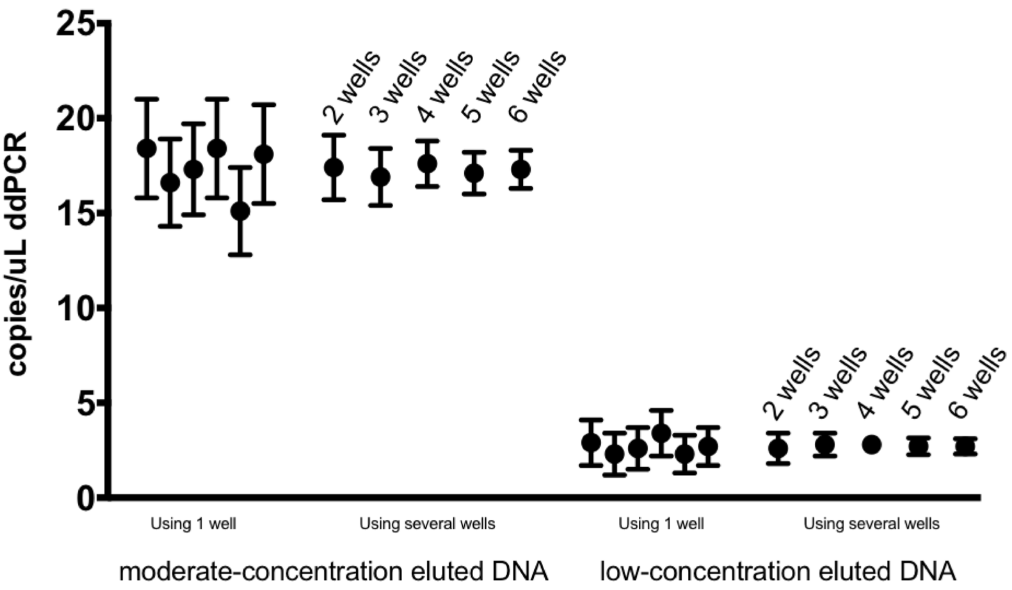

Supplementary Figure 3: Impact on the number of wells used for analysis on confidence intervals: reducing the number of wells to 3 allows a 20% standard deviation, acceptable for absolute plasmatic cfDNA quantification.

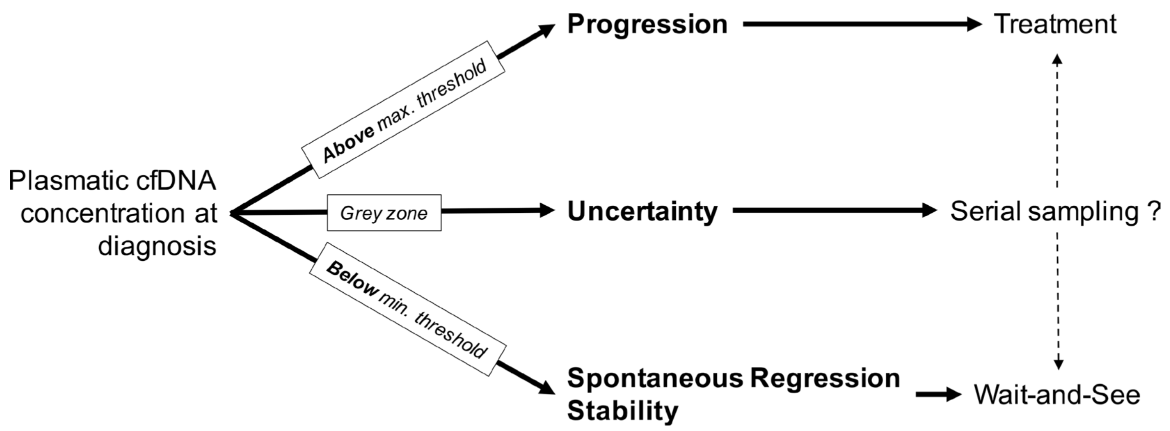

Supplementary Figure 4: Proposition of the possible integration of the measurement of cfDNA in the decision making and management of desmoid tumors during the wait-and-see strategy.

Supplementary Table 1: Raw data. Supplementary_Table_1

Supplementary Table 2 (T2) : Diagnostic performance table of cfDNA concentration at the time of diagnosis for the prediction of regressive & stable desmoids: lower threshold. Supplementary_Table_2

Supplementary Table 3 (T3) : Diagnostic performance table of cfDNA concentration at the time of diagnosis for the prediction of regressive & stable desmoids: upper threshold. Supplementary_Table_3

Supplementary Table (T4): Clinico-pathological characteristics of the 6 patients with a CTNNB1 mutation detectable on plasmatic cfDNA

Sex	Age	Multiple DT	Topography	Size (mm)	Evolution	Follow-up (months)
F	27	-	Abdominal wall	25	Stable	8
M	64	-	Thorax	100	Progressive	8
F	54	-	Upper arm	25	Stable	19
F	55	+	Thorax	57	Self-Regressive	3
F	42	-	Abdominal wall	25	Stable	2
F	32	-	Intra-abdominal	60	Progressive	10

Supplementary table 5 (T5): cfDNA concentrations in six healthy patients

Age	plasmatic concentration of cfDNA	
	(copies/µL of ddPCR)	(copies/mL of plasma)
42	10,5	625
23	7,3	487,5
22	5,3	331,25
32	6,7	418,75
29	6,6	412,5
28	4,33	270,625
