# Supplementary material for: Proof of concept: prognostic value of the plasmatic concentration of circulating cell free DNA in desmoid tumors using ddPCR
Source: Oncotarget. 2018 Apr 6;9(26):18296–308. doi: 10.18632/oncotarget.24817 (PMC5915073; doi:10.18632/oncotarget.24817)
Supplement: Supplementary file 3 [file oncotarget-09-18296-s003.docx]

**Supplementary table 2 (ST2) :** Diagnostic performance table of cfDNA concentration at the time of diagnosis for the prediction of regressive & stable desmoids: lower threshold.

| **copies/mL plasma  (positive test > cutoff)** | **Sensitivity** | **95% CI** | **Specificity** | **95% CI** | **Likelihood ratio** | **True positive** | **True negative** | **False positive** | **False negative** |
| --- | --- | --- | --- | --- | --- | --- | --- | --- | --- |
| **< 94.59** | **4,167** | **0,1054% - 21,12%** | **100** | **59,04% - 100%** | **/** | **1** | **7** | **23** | **0** |
| **< 105.4** | **8,333** | **1,026% - 27%** | **100** | **59,04% - 100%** | **/** | **2** | **7** | **22** | **0** |
| **< 127.5** | **12,5** | **2,656% - 32,36%** | **100** | **59,04% - 100%** | **/** | **3** | **7** | **21** | **0** |
| **< 152.9** | **16,67** | **4,735% - 37,38%** | **100** | **59,04% - 100%** | **/** | **4** | **7** | **20** | **0** |
| **< 200.4** | **20,83** | **7,132% - 42,15%** | **100** | **59,04% - 100%** | **/** | **5** | **7** | **19** | **0** |
| **< 271.3** | **25** | **9,773% - 46,71%** | **100** | **59,04% - 100%** | **/** | **6** | **7** | **18** | **0** |
| **< 312.5** | **29,17** | **12,62% - 51,09%** | **100** | **59,04% - 100%** | **/** | **7** | **7** | **17** | **0** |
| **< 331.3** | **33,33** | **15,63% - 55,32%** | **100** | **59,04% - 100%** | **/** | **8** | **7** | **16** | **0** |
| **< 346.3** | **37,5** | **18,8% - 59,41%** | **100** | **59,04% - 100%** | **/** | **9** | **7** | **15** | **0** |
| **< 431.7** | **41,67** | **22,11% - 63,36%** | **100** | **59,04% - 100%** | **/** | **10** | **7** | **14** | **0** |
| **< 575** | **45,83** | **25,55% - 67,18%** | **100** | **59,04% - 100%** | **/** | **11** | **7** | **13** | **0** |
| **< 679.2** | **50** | **29,12% - 70,88%** | **100** | **59,04% - 100%** | **/** | **12** | **7** | **12** | **0** |
| **< 754.2** | **54,17** | **32,82% - 74,45%** | **100** | **59,04% - 100%** | **/** | **13** | **7** | **11** | **0** |
| **< 800** | **58,33** | **36,64% - 77,89%** | **100** | **59,04% - 100%** | **/** | **14** | **7** | **10** | **0** |
| **< 845.8** | **62,5** | **40,59% - 81,2%** | **100** | **59,04% - 100%** | **/** | **15** | **7** | **9** | **0** |
| **< 900** | **66,67** | **44,68% - 84,37%** | **100** | **59,04% - 100%** | **/** | **16** | **7** | **8** | **0** |
| < 933.3 | 66,67 | 44,68% - 84,37% | 85,71 | 42,13% - 99,64% | 4,667 | 16 | 6 | 8 | 1 |
| < 970.8 | 70,83 | 48,91% - 87,38% | 85,71 | 42,13% - 99,64% | 4,958 | 17 | 6 | 7 | 1 |
| < 979.2 | 75 | 53,29% - 90,23% | 85,71 | 42,13% - 99,64% | 5,25 | 18 | 6 | 6 | 1 |
| < 987.5 | 79,17 | 57,85% - 92,87% | 85,71 | 42,13% - 99,64% | 5,542 | 19 | 6 | 5 | 1 |
| < 1038 | 79,17 | 57,85% - 92,87% | 71,43 | 29,04% - 96,33% | 2,771 | 19 | 5 | 5 | 2 |
| < 1100 | 83,33 | 62,62% - 95,26% | 71,43 | 29,04% - 96,33% | 2,917 | 20 | 5 | 4 | 2 |
| < 1121 | 87,5 | 67,64% - 97,34% | 71,43 | 29,04% - 96,33% | 3,063 | 21 | 5 | 3 | 2 |
| < 1138 | 87,5 | 67,64% - 97,34% | 57,14 | 18,41% - 90,1% | 2,042 | 21 | 4 | 3 | 3 |
| < 1229 | 91,67 | 73% - 98,97% | 57,14 | 18,41% - 90,1% | 2,139 | 22 | 4 | 2 | 3 |
| < 1375 | 100 | 85,75% - 100% | 57,14 | 18,41% - 90,1% | 2,333 | 24 | 4 | 0 | 3 |
| < 1613 | 100 | 85,75% - 100% | 42,86 | 9,899% - 81,59% | 1,75 | 24 | 3 | 0 | 4 |
| < 1829 | 100 | 85,75% - 100% | 28,57 | 3,669% - 70,96% | 1,4 | 24 | 2 | 0 | 5 |
| < 1917 | 100 | 85,75% - 100% | 14,29 | 0,361% - 57,87% | 1,167 | 24 | 1 | 0 | 6 |
