# Supplementary material for: Proof of concept: prognostic value of the plasmatic concentration of circulating cell free DNA in desmoid tumors using ddPCR
Source: Oncotarget. 2018 Apr 6;9(26):18296–308. doi: 10.18632/oncotarget.24817 (PMC5915073; doi:10.18632/oncotarget.24817)
Supplement: Supplementary file 4 [file oncotarget-09-18296-s004.docx]

**Supplementary table 3 (ST3) :** Diagnostic performance table of cfDNA concentration at the time of diagnosis for the prediction of regressive & stable desmoids: upper threshold.

| copies/mL plasma  (Positive test > cutoff) | Sensitivity | 95% CI | Specificity | 95% CI | Likelihood ratio | True positive | True negative | False positive | False negative |
| --- | --- | --- | --- | --- | --- | --- | --- | --- | --- |
| > 94.59 | 100 | 59,04 - 100% | 4,167 | 0,1054% - 21,12% | 1,043 | 7 | 1 | 0 | 23 |
| > 105.4 | 100 | 59,04% - 100% | 8,333 | 1,026% - 27% | 1,091 | 7 | 2 | 0 | 22 |
| > 127.5 | 100 | 59,04% - 100% | 12,5 | 2,656% - 32,36% | 1,143 | 7 | 3 | 0 | 21 |
| > 152.9 | 100 | 59,04% - 100% | 16,67 | 4,735% - 37,38% | 1,2 | 7 | 4 | 0 | 20 |
| > 200.4 | 100 | 59,04% - 100% | 20,83 | 7,132% - 42,15% | 1,263 | 7 | 5 | 0 | 19 |
| > 271.3 | 100 | 59,04% - 100% | 25 | 9,773% - 46,71% | 1,333 | 7 | 6 | 0 | 18 |
| > 312.5 | 100 | 59,04% - 100% | 29,17 | 12,62% - 51,09% | 1,412 | 7 | 7 | 0 | 17 |
| > 331.3 | 100 | 59,04% - 100% | 33,33 | 15,63% - 55,32% | 1,5 | 7 | 8 | 0 | 16 |
| > 346.3 | 100 | 59,04% - 100% | 37,5 | 18,8% - 59,41% | 1,6 | 7 | 9 | 0 | 15 |
| > 431.7 | 100 | 59,04% - 100% | 41,67 | 22,11% - 63,36% | 1,714 | 7 | 10 | 0 | 14 |
| > 575 | 100 | 59,04% - 100% | 45,83 | 25,55% - 67,18% | 1,846 | 7 | 11 | 0 | 13 |
| > 679.2 | 100 | 59,04% - 100% | 50 | 29,12% - 70,88% | 2 | 7 | 12 | 0 | 12 |
| > 754.2 | 100 | 59,04% - 100% | 54,17 | 32,82% - 74,45% | 2,182 | 7 | 13 | 0 | 11 |
| > 800 | 100 | 59,04% - 100% | 58,33 | 36,64% - 77,89% | 2,4 | 7 | 14 | 0 | 10 |
| > 845.8 | 100 | 59,04% - 100% | 62,5 | 40,59% - 81,2% | 2,667 | 7 | 15 | 0 | 9 |
| > 900 | 100 | 59,04% - 100% | 66,67 | 44,68% - 84,37% | 3 | 7 | 16 | 0 | 8 |
| > 933.3 | 85,71 | 42,13% - 99,64% | 66,67 | 44,68% - 84,37% | 2,571 | 6 | 16 | 1 | 8 |
| > 970.8 | 85,71 | 42,13% - 99,64% | 70,83 | 48,91% - 87,38% | 2,939 | 6 | 17 | 1 | 7 |
| > 979.2 | 85,71 | 42,13% - 99,64% | 75 | 53,29% - 90,23% | 3,429 | 6 | 18 | 1 | 6 |
| > 987.5 | 85,71 | 42,13% - 99,64% | 79,17 | 57,85% - 92,87% | 4,114 | 6 | 19 | 1 | 5 |
| > 1038 | 71,43 | 29,04% - 96,33% | 79,17 | 57,85% - 92,87% | 3,429 | 5 | 20 | 2 | 4 |
| > 1100 | 71,43 | 29,04% - 96,33% | 83,33 | 62,62% - 95,26% | 4,286 | 5 | 21 | 2 | 3 |
| > 1121 | 71,43 | 29,04% - 96,33% | 87,5 | 67,64% - 97,34% | 5,714 | 5 | 22 | 2 | 2 |
| > 1138 | 57,14 | 18,41% - 90,1% | 87,5 | 67,64% - 97,34% | 4,571 | 4 | 22 | 3 | 2 |
| > 1229 | 57,14 | 18,41% - 90,1% | 91,67 | 73% - 98,97% | 6,857 | 4 | 23 | 3 | 1 |
| **> 1375** | **57,14** | **18,41% - 90,1%** | **100** | **85,75% to 100%** | **/** | **4** | **24** | **3** | **0** |
| **> 1613** | **42,86** | **9,899% - 81,59%** | **100** | **85,75% to 100%** | **/** | **3** | **24** | **4** | **0** |
| **> 1829** | **28,57** | **3,669% - 70,96%** | **100** | **85,75% to 100%** | **/** | **2** | **24** | **5** | **0** |
| **> 1917** | **14,29** | **0,361% - 57,87%** | **100** | **85,75% to 100%** | **/** | **1** | **24** | **6** | **0** |
